# Machine Learning for the Preliminary Diagnosis of Dementia

**DOI:** 10.1155/2020/5629090

**Published:** 2020-03-07

**Authors:** Fubao Zhu, Xiaonan Li, Haipeng Tang, Zhuo He, Chaoyang Zhang, Guang-Uei Hung, Pai-Yi Chiu, Weihua Zhou

**Affiliations:** 1 School of Computer and Communication Engineering, Zhengzhou University of Light Industry, Zhengzhou, Henan, USA; 2 School of Computing Sciences and Computer Engineering, University of Southern Mississippi, Hattiesburg, MS, USA; 3 College of Computing, Michigan Technological University, Houghton, MI, USA; 4 Department of Nuclear Medicine, Chang Bing Show Chwan Memorial Hospital, Changhua, Taiwan; 5 Department of Neurology, Show Chwan Memorial Hospital, Changhua, Taiwan

## Abstract

**Objective.:**

The reliable diagnosis remains a challenging issue in the early stages of dementia. We aimed to develop and validate a new method based on machine learning to help the preliminary diagnosis of normal, mild cognitive impairment (MCI), very mild dementia (VMD), and dementia using an informant-based questionnaire.

**Methods.:**

We enrolled 5,272 individuals who filled out a 37-item questionnaire. In order to select the most important features, three different techniques of feature selection were tested. Then, the top features combined with six classification algorithms were used to develop the diagnostic models.

**Results.:**

Information Gain was the most effective among the three feature selection methods. The Naive Bayes algorithm performed the best (accuracy = 0.81, precision = 0.82, recall = 0.81, and *F*-measure = 0.81) among the six classification models.

**Conclusion.:**

The diagnostic model proposed in this paper provides a powerful tool for clinicians to diagnose the early stages of dementia.

## 1. Introduction

Alzheimer’s disease and other dementias that occur most frequently in older adults are heavy burdens on families and society due to their highly intellectual disability. To date, there is no effective treatment to slow down or stop the progression of dementia. It is critical to focus on the early stages, timely intervention, and delay of the disease. The clinical diagnosis of dementia is based on the detailed medical history provided by patients and their families, neurological examination, and neuropsychological tests. Other tests including hematology, CT, and MRI should be performed to rule out other causes of dementia. Neuropsychological tests play a crucial role in detecting dysfunctions in human “cognitive domains.” Even though there have been several clinical measures for the early diagnosis of dementia, a lot of subjectivity still exists [1–3]. It is of great importance to develop better diagnostic tools.

Accurate classification of cognitive impairment is not only beneficial to individuals but also important for medicine. In clinical diagnosis, it is time-intensive for the manual diagnosis of cognitive impairment, which may require multiple pieces of information like a neuropsychological test score, laboratory study results, knowledgeable informant reports, and so on. The efficiency and accuracy of the diagnosis are determined by the professional level of the practitioner. In several remote areas lacking professional personnel, it will be a much more difficult task for classification and the early diagnosis of dementia. Machine Learning is an advanced computing technology which can improve the analysis of medical data and automatically make the diagnostic decision [4].

The aims of the paper were (1) to optimize or even reduce the number of neuropsychological tests used to classify dementia patients by using feature selection algorithms and (2) to develop and validate an accurate classification model based on the diagnostic information of enrolled subjects.

## 2. Materials and Methods

**Figure 1:**
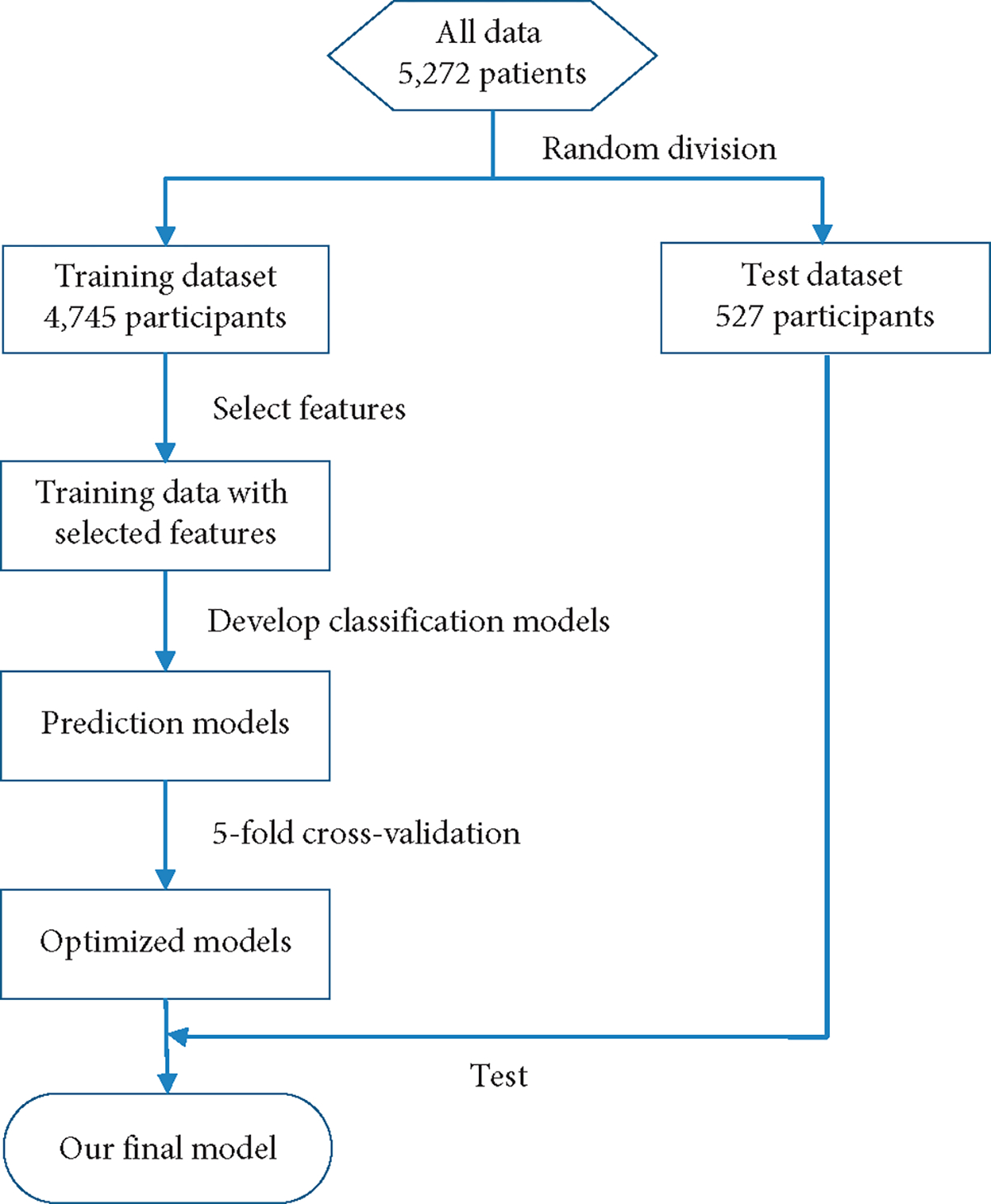
Flow chart of data processing in our method to develop and validate the diagnosis model.

The participants were selected from the register-based database of the Show Chwan Health System. The study design was retrospective, and the data were analyzed anonymously. The Medical Research Ethics Committee of Show Chwan Memorial Hospital (Show Chwan IRB number: 1041208) reviewed the project, and the Data Inspectorate approved the study [5]. Figure 1 shows the workflow of our method. The dataset was first randomly split into a training dataset and a test dataset. Feature selection, model optimization, and 5-fold cross-validation were applied to the training data to develop and optimize the diagnosis models. Finally, the models were tested with the test data to find the optimal diagnosis model.

### 2.1. Participants.

We followed the method of Sun et al. [6]. Clinical data of a total of 5,272 patients were analyzed. Normal cognition (NC), MCI, VMD, or dementia were defined as follows: NC referred to individuals who did not meet criteria for any of the conditions listed in the National Institute on Aging-Alzheimer’s Association (NIA-AA) core clinical criteria for all-cause dementia [7] and had a clinical dementia ratings (CDR) score of 0 [8]. MCI was defined as the individuals who had cognitive change with impairment in the domains of orientation and/or judgment but without impairment in social or occupational functioning and had a CDR score of 0.5 [9]. In addition, at least one cognitive domain in CASI adjusted with age and education level should be impaired [10, 11]. In the domains of community affairs, home hobbies, and personal care, the CDR should be 0. VMD was defined as the individuals who met the NIA-AA criteria for all-cause dementia with a CDR score of 0.5 [7], had mild impairment in 2 or more cognitive domains, and had mild decline in daily functions, including the domains of community affairs, home hobbies, or personal care in which the CDR should be ≥0.5. The definition of all-cause dementia was based on the core clinical criteria recommended by the NIA-AA [7]. The different types of dementia were diagnosed according to each consensus criterion.

A structured clinical history was taken from the participant and the principal caregiver. The clinical history was taken to detect any subtle change of behavior or personality and any mental decline from previous levels of functioning and to determine whether this decline interfered with the ability to function at work or in routine activities. In addition to the history of cognitive status, objective assessments including the CDR, Cognitive Abilities Screening Instrument (CASI), and Montreal Cognitive Assessment (MoCA) were performed to evaluate memory, executive function, orientation, visual-spatial ability, and language function. The severity of dementia was then determined by the CDR. Daily function was assessed with the Instrumental Activities of Daily Living (IADL) scale [9]. Neuropsychiatric Inventory (NPI) was used to assess the neuropsychiatric symptoms of participants [12]. The scores of CASI and MOCA were evaluated as the outcome of the diagnostic models in this study.

The enrolled participants were randomly divided into a training set (4,745 participants) to build the diagnostic models and an independent test set (527 participants) to validate the diagnostic models in discriminating normal, MCI, VMD, and dementia. In order to estimate the generalization error, this procedure was repeated 5 times independently to avoid any deviation caused by randomly partitioning data sets. We selected a set of training sets and test sets whose category distribution was similar to the situation in the actual data, which is similar to the stratified sampling technique. In the training set, there were 328 for normal, 1,234 for MCI, 718 for VMD, and 2,465 for dementia. In the test set, there were 51 for normal, 113 for MCI, 98 for VMD, and 265 for dementia. In the diagnosis of cognitive disorders, neurosurgeons interviewed the study subjects through a standardized neurological examination, and historical inquiry fully grasped the subject’s memory complaints and clinical manifestations and completed the CDR score. A diagnostic team was composed of physicians in the neurology department of cognitive impairment. The results of the neurological examination, history, and neuropsychological tests of each study were evaluated. Finally, the diagnosis was given. Informed consent has been received from all participants.

### 2.2. Feature Selection.

In machine learning, 37 features have potentially possessed different importance in the diagnosis of dementia. Feature selection can effectively eliminate redundant and/or unrelated features. On the one hand, it can improve the generalization performance and efficiency of the machine learning algorithm; on the other hand, it can simplify the procedure of diagnosis and enhance the practicality in the clinic. In this section, we explored three feature selection methods, which are Random Forest, Information Gain, and Relief.

#### 2.2.1. The Random Forest Algorithm for Feature Selection.

We can use the Random Forest model to filter features and get their correlation with classification. Due to the inherent randomness of Random Forest, the model may give a different weight of importance each time. However, when training the model for several runs, in each run, we select a certain number of characteristics and retain the intersection between the new feature set and the set of features selected in other runs. After a certain number of runs, we can finally get a certain amount of features. Then, we calculate the out-of-bag error rate corresponding to these features and use the feature set with the lowest out-of-bag error rate as the last selected feature set. This method was implemented in the machine learning software package by Python [13]. The feature selection process with the Random Forest algorithm is illustrated in Algorithm 1.

#### 2.2.2. The Information Gain Algorithm for Feature Selection.

Information Gain is an effective method for feature selection. In the Information Gain, the criterion is to measure how much information the feature can bring to the classification model, and the more information it brings, the more significant it is. Information Gain is based on the theory of entropy, which has been widely used by researchers in various application scenarios. The entropy is a notation in information theory, which can be applied to evaluate the importance of features. The classic formula for Shannon entropy is $H(x)=-\sum_{i=1}^{n}\;p\left(x_{i}\right)log\left(p\left(x_{i}\right)\right)$, where $p\left(x_{i}\right)$ is the probability density function estimated with a Gaussian kernel. We used the Information Gain algorithm implemented in Weka, which is a powerful open-source Java-based machine learning workbench. Based on the Information Gain score, the features with score values below a threshold were filtered out.

#### 2.2.3. The Relief Algorithm for Feature Selection.

The core idea of Relief is that a good feature should make the eigenvalues of the nearest neighbor samples be the same or similar and make the values between different classes of nearest neighbors differ or differ greatly. The advantages of the Relief algorithm are high operation efficiency, no restriction on data type, and insensitivity to relations among features. The drawback of the Relief algorithm is that, unlike many feature evaluation algorithms, such as Information Gain, the Relief algorithm cannot remove redundant features, and the algorithm will give all kinds of high correlation features, regardless of whether the feature is redundant with other features. We used the implementation of the Relief algorithm available in Weka.

### 2.3. Construction of the Diagnostic Models.

We examined six different classification algorithms to build the diagnostic models, including Random Forest, AdaBoost, LogitBoost, Neural Network (NN), Naive Bayes, and Support Vector Machine (SVM). To optimize the corresponding model parameters and to estimate the performance, we used the Scikit-learn Python toolbox and the experimental mode (Experimenter) in Weka, which allows large-scale experiments to run with results stored in a database for later retrieval and analysis. Moreover, the accuracy, precision, recall, and *F*-measure as performance metrics were computed to evaluate the diagnostic models using the test set. The diagnostic models’ training and parameter optimization were done by 5-fold cross-validation.

Random Forest is a classifier with multiple decision trees, in which the output is determined by a majority vote of the trees. It is not sensitive to noise or overtraining, because resampling is not based on weighting. It has relatively high accuracy and computational efficiency. AdaBoost and LogitBoost are boosting algorithms in which the key idea is to train different classifiers (weak classifiers) for the same training set and then combine these weak classifiers to form a stronger final classifier (strong classifier). We used the Multilayer Perceptron (MLP) as an NN implementation, which is a forward-structured Artificial Neural Network that maps a set of input vectors to a set of output vectors. Naive Bayesian is a classification method based on Bayes theorem and characteristic conditionally independent hypothesis. SVM searches for the best separated hyperplane as the maximum marginal hyperplane to solve the problem of multiclass classification.

## 3. Results

The detailed demographical data of the test group are shown in Table 1. The results demonstrated that the cognitive function, the function of activities of daily living, and the severity of neuropsychiatric symptoms deteriorated as the stages of dementia increased.

### 3.1. Feature Selection

#### 3.1.1. Feature Ranking.

**Figure 2:**
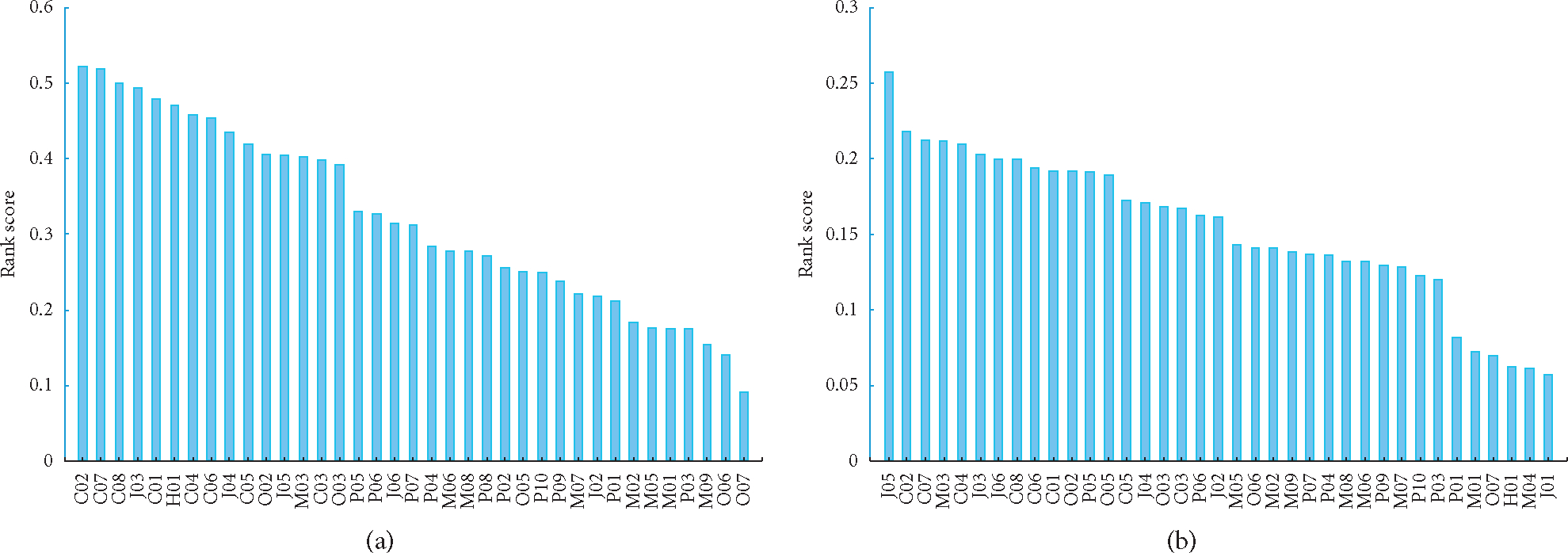

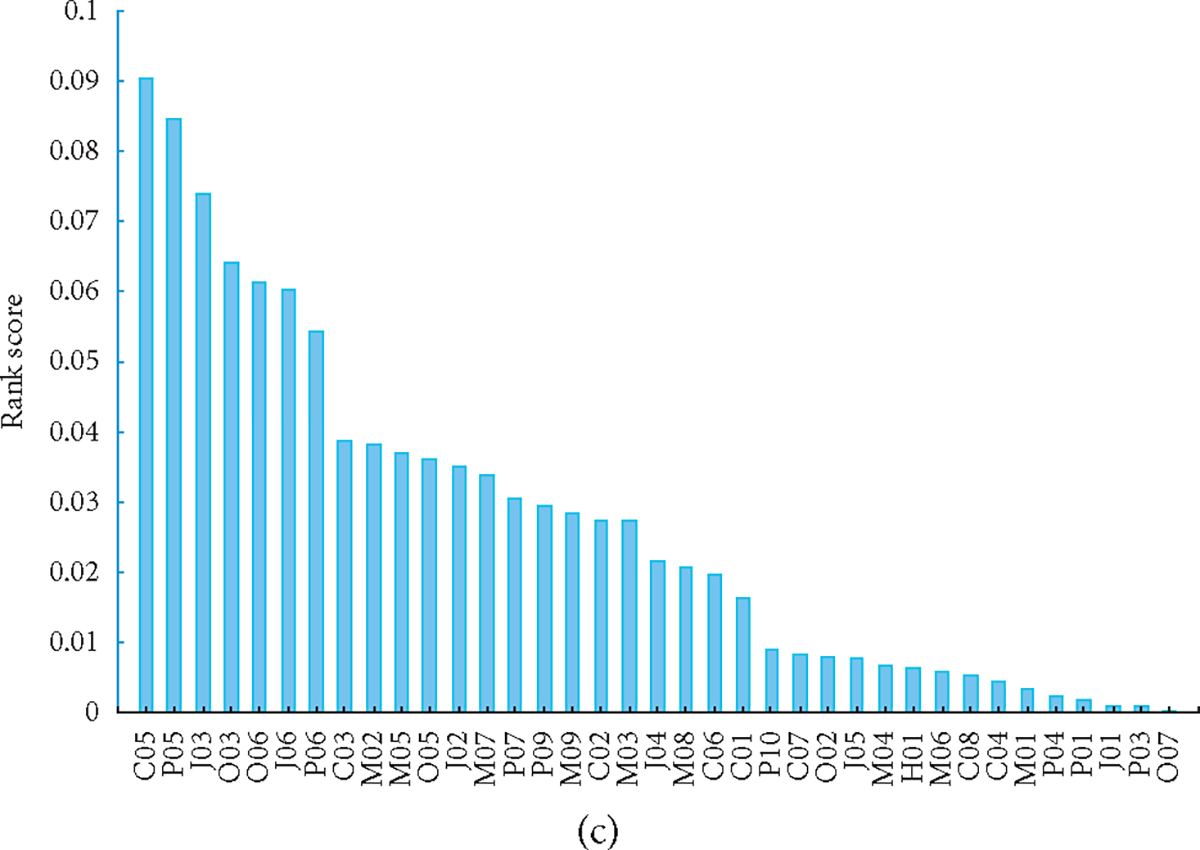
Feature ranking by the (a) Information Gain, (b) Relief algorithms, and (c) Random Forest.

Figure 2 shows the feature ranking. Figure 2(a) shows the features ordered by their rank score in the Information Gain algorithm, Figure 2(b) shows the features ordered by their rank score in the Relief algorithm, and Figure 2(c) shows the features ordered by their rank score in the Random Forest algorithm.

#### 3.1.2. Features Selection.

**Figure 3:**
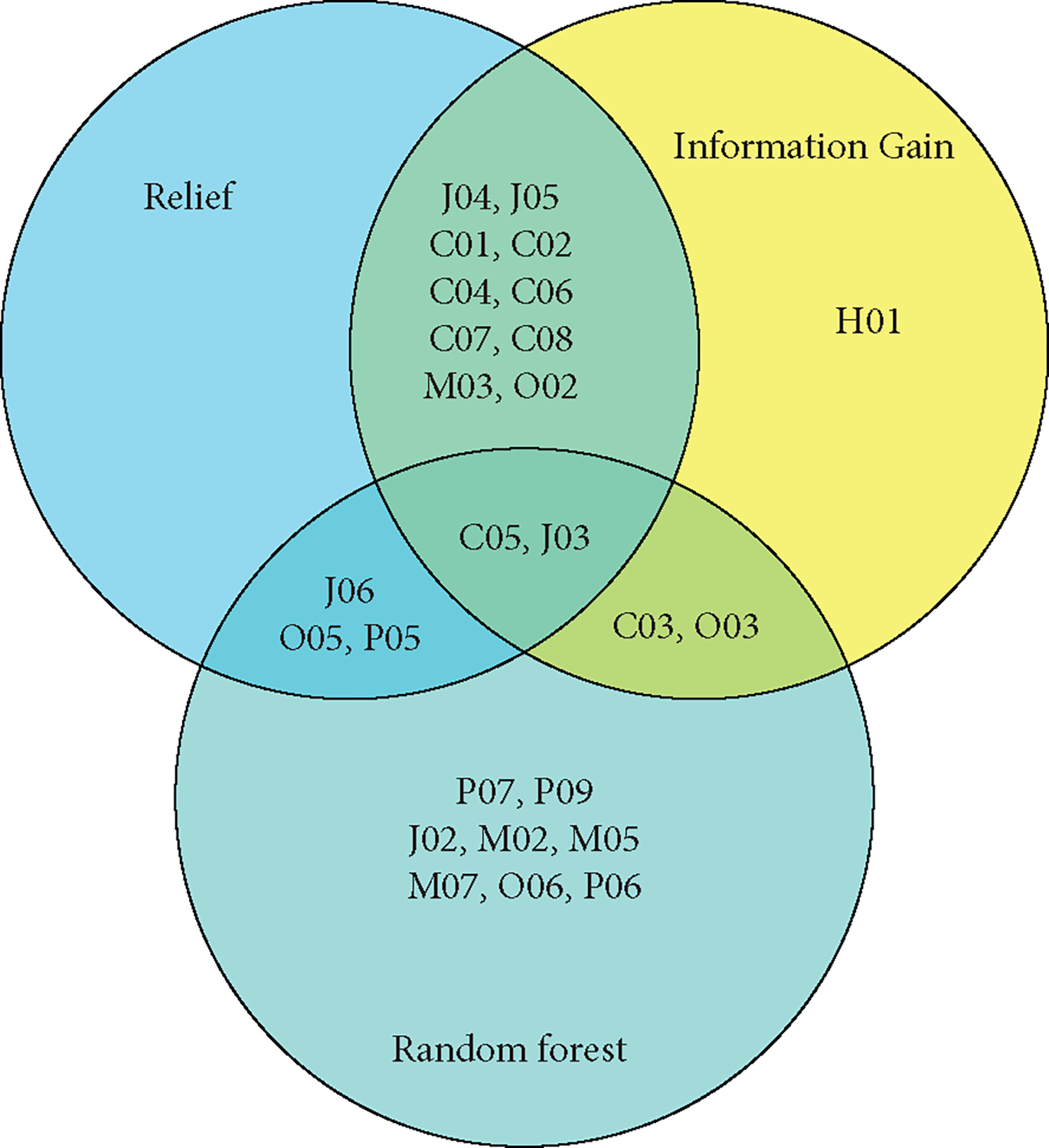
The top 15 features selected by the Random Forest, Information Gain, and Relief algorithms.

Figure 3 shows the top 15 features selected according to the feature selection algorithm. The top 15 features selected by the three feature selection algorithms were different. Among the features selected by the Random Forest, there were 5 features common with the features by the Information Gain, 4 features common with those by Relief, and 2 features common with those by Information Gain. Among the features selected by Information Gain, there were 12 features common with those by Relief.

**Algorithm 1:** The Random Forest algorithm for feature selection.

	Input: A training set: ${\left\{x_{i},y_{i}\right\}}_{i=1}^{n},\;x_{i}\in X,\;y_{i}\in \{0,\;1,\;2,\;3\}$, i=1,2,…,n
	where n is the size of the training set, $x_{i}$ denotes the features in the sample, $y_{i}$ denotes the class label in the sample, and X denotes the feature space
	Output: The key feature T;
	Begin
(1)	Set all the feature weights is 0, T is empty;
(2)	for *i* = 1 to m do;
(3)	Given a tree ensemble model
(4)	Computes the importance of each feature.
	Average over several randomized trees:
	Importance (feature *t*) = sum (over nodes which split on feature *t*) of the gain, where gain is scaled by the number of instances passing through node,
	Normalize importance for tree to sum to 1.
	Normalize feature important vector to sum to 1.
(5)	T = the intersection of the set $t_{i-1}$ of the set of $t_{i}$.
	End

### 3.2. Optimization of Diagnostic Models.

We use grid-SearchCV to optimize the parameters of the model. The optimal model parameters are shown in Table 2. The default parameters of the algorithm are not displayed.

### 3.3. Evaluation of Diagnostic Performance.

Table 3 shows the classification performance of six algorithms when using all the 37 features. The accuracy, precision, recall, and *F*-measure are reported. The Naive Bayes algorithm performed the best (accuracy = 0.87, precision = 0.88, recall = 0.87, and *F*-measure = 0.87) among the six classification models, followed by the MLP (accuracy = 0.87, precision = 0.87, recall = 0.87, and *F*-measure = 0.87) and SVM (accuracy = 0.87, precision = 0.86, recall = 0.87, and *F*-measure = 0.86).

Table 4 shows the classification performance of six algorithms under three feature selections. The Naive Bayes algorithm performed the best (accuracy = 0.81, precision = 0.82, recall = 0.81, and *F*-measure = 0.81) among the six classification models, followed by the Random Forest (accuracy = 0.78, precision = 0.79, recall = 0.78, and *F*-measure = 0.78) and LogitBoost algorithm (accuracy = 0.76, precision = 0.77, recall = 0.76, and *F*-measure = 0.74).

Table 5 shows the results of diagnosing normal, MCI, VMD, and dementia by the six classification models. The results of Random Forest, AdaBoost, and Naïve Bayes were obtained using the Information Gain feature selection; the results of LogitBoost and MLP were obtained using the Random Forest feature selection; the results of SVM were obtained using the Relief feature selection. The Naive Bayes algorithm effectively improved the overall performance in classifying normal (sensitivity = 0.84, specificity = 0.94), MCI (sensitivity = 0.62, specificity = 0.93), VMD (sensitivity = 0.72, specificity = 0.93), and dementia (sensitivity = 0.92, specificity = 0.95).

**Figure 4:**
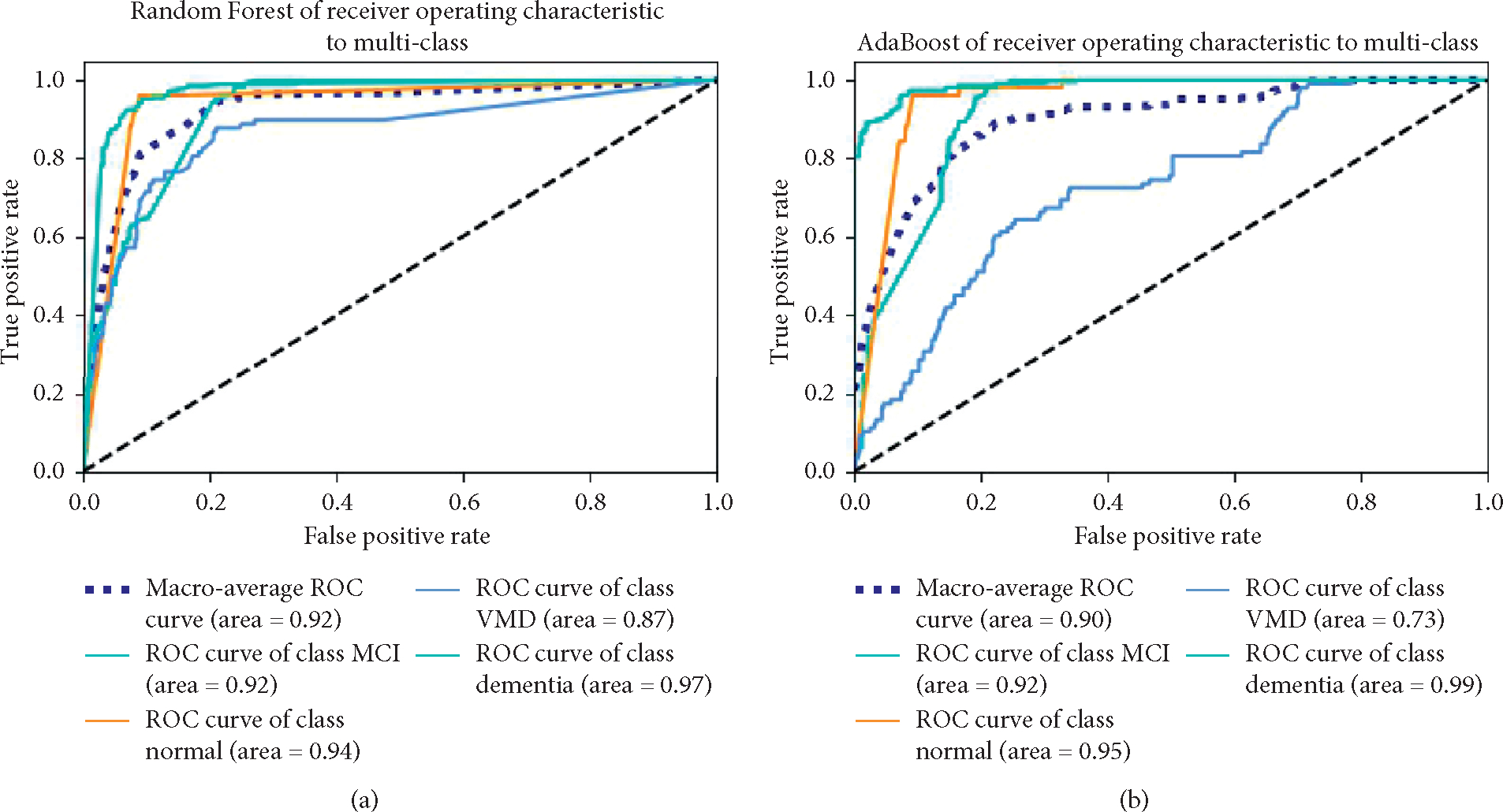

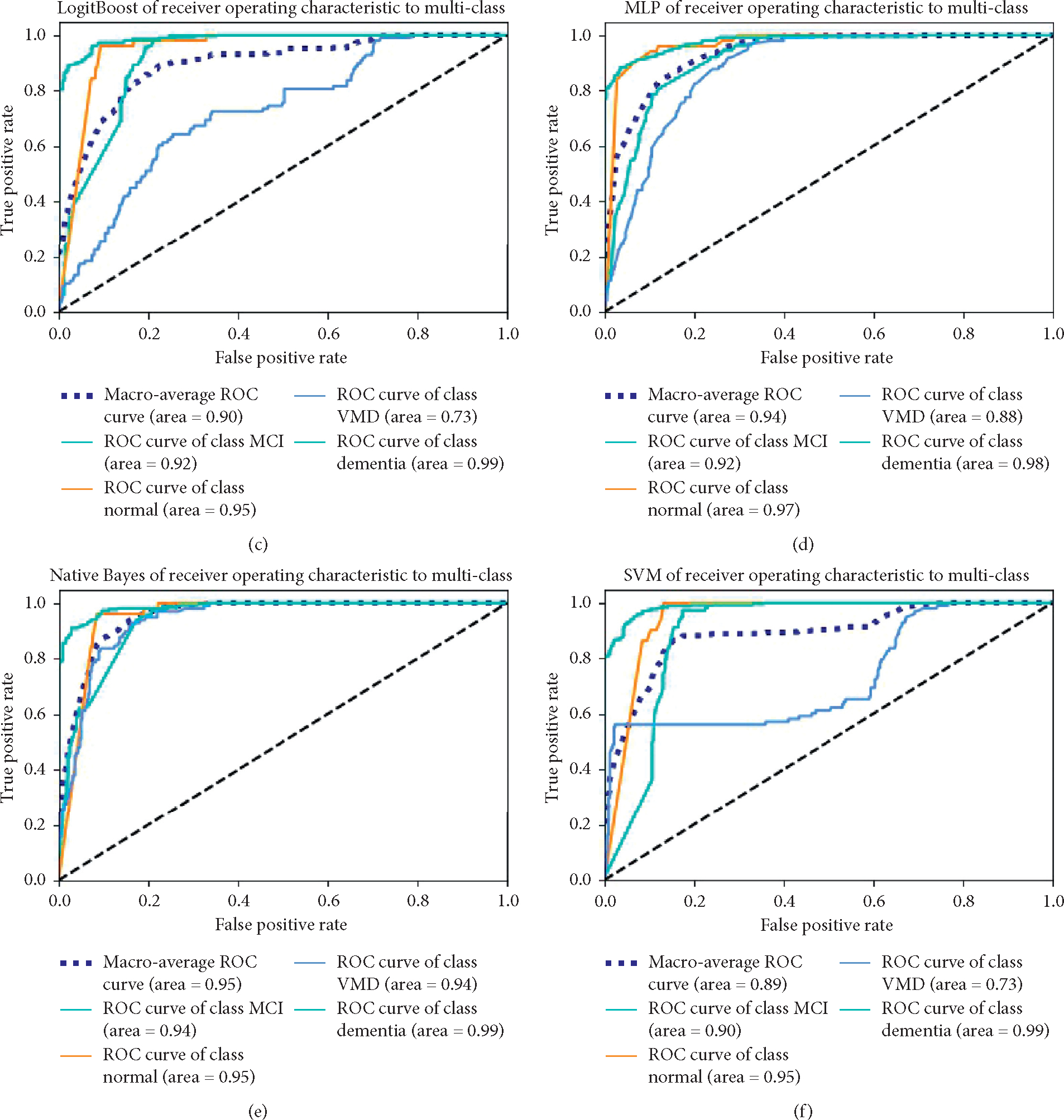
The ROC curve analysis of the diagnostic models. (a) Random Forest. (b) AdaBoost. (c) LogitBoost. (d) MLP. (e) Naive Bayes. (f) SVM.

Figure 4 shows the receiver operating characteristic (ROC) analysis of diagnosing normal, MCI, VMD, and dementia by the six classification models. The Naive Bayes algorithm performed the best among the six classification models. The area under the ROC curve (AUC) is 0.95.

**Figure 5:**
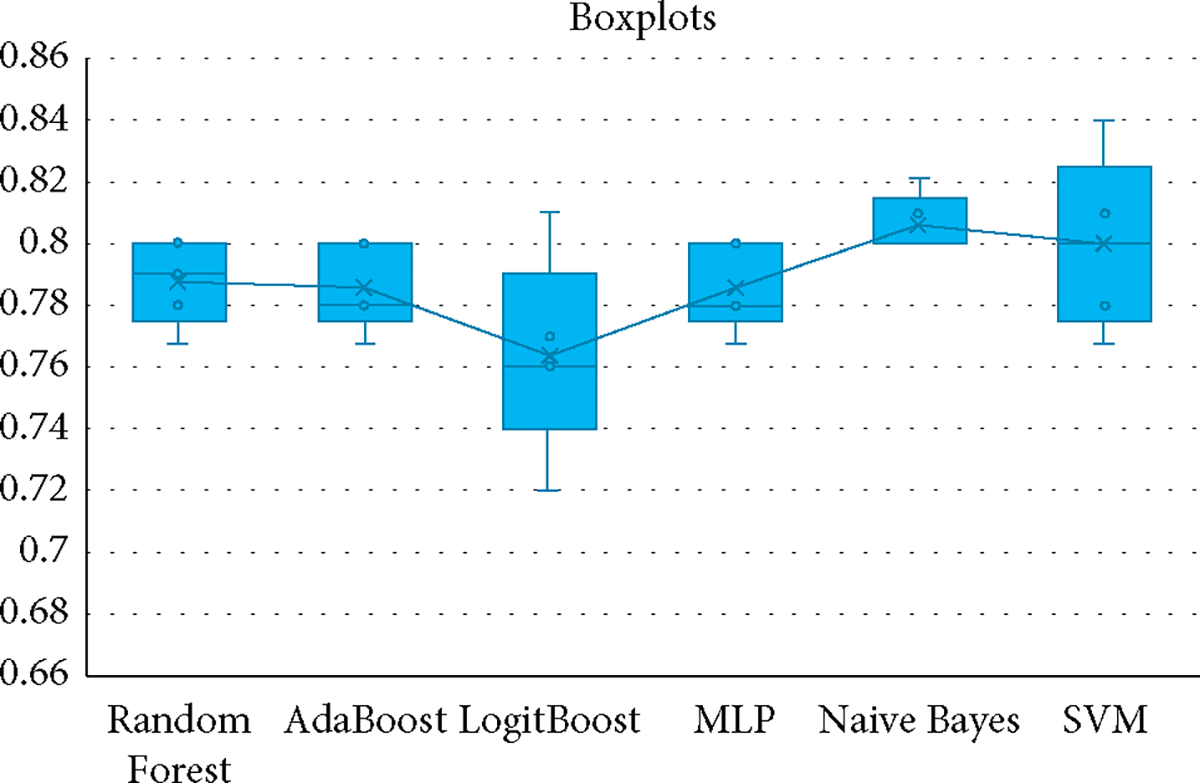
The result obtained for each algorithm in the 5 rounds.

Figure 5 shows the results of 5-fold cross-validation obtained for each algorithm in the 5 rounds.

## 4. Discussion

The purpose of this study was to provide a new clinical tool based on machine learning for the early diagnosis of dementia. To find an optimal classification model, we compared different feature selection algorithms and classification algorithms using the same data. We carried out a sensitivity analysis for testing the robustness of the results by our classification algorithms. Our results demonstrated that, in feature selection, Information Gain performed the best among the three feature selection algorithms in the six classification models. Random Forest as a feature selection algorithm makes the rare classes (normal) easy to classify correctly. Among the classification models, the Naive Bayes algorithm performs the best, followed by the Random Forest and LogitBoost algorithm.

Although several studies have constructed diagnostic models, to our knowledge, current screening tools have great limitations in class imbalance problems and clinical applicability. Class imbalance [14–16] exists in many real-world decision making problems. In this paper, the ensemble learning technique used in Random Forest, AdaBoost, and LogitBoost can increase the accuracy of a single classifier by combining the classification results from different trained classifiers; it has been demonstrated to increase the performance when processing the imbalance problem [17]. Naïve Bayes classifier deals with class imbalance naturally by multiplying the likelihood by the class prior probability. In SVM, the classes with fewer samples have higher misclassification penalty, which can alleviate the imbalance. Nevertheless, the accuracy of our diagnostic model still remains scope for improvement.

Several studies [18–20] have achieved promising results for clinical applicability. Bron et al. [18] organized a grand challenge that aimed to objectively compare algorithms based on a clinically representative multicenter data set. This challenge provided insight into the best strategies for computer-aided diagnosis of dementia. Amoroso et al. [19] use MRI data from the Parkinson’s Progression Markers Initiative (PPMI) to extract imaging markers and learn an accurate classification model. Heister et al. [20] predicted MCI outcome with clinically available MRI and CSF biomarkers. However, these methods had limitations on clinical applicability. Clinical applicability issues also existed in our study. In this paper, we compared three different feature selection algorithms in order to choose the best feature selection algorithm. However, it can be seen from the results that the top 15 features selected by the three feature selection algorithms are different. The features C05 and J03 are selected by three feature selection algorithms at the same time. Random Forest feature selection algorithm and other two feature selection algorithms have few common features, but 12 features selected by information gain and relief feature selection algorithm are the same. The information contained in the 37 features is different, and how to pick out features that are more valuable for classification is still a problem that needs to be studied. Our future work will further explore sampling techniques and classification algorithms to improve our diagnostic model.

## 5. Limitations

The study was conducted in only three hospitals in Taiwan, which may show selection bias. More medical centers and subjects are needed to validate our method further.

## 6. Conclusions

We developed and validated new approaches to diagnosing normal, MCI, VMD, and dementia. As a result, Information Gain was the most effective for feature selection among the three feature selection methods. Random Forest improved the overall performance of all diagnostic models. Among the six classification models, the Naive Bayes algorithm performed the best (accuracy = 0.81, precision = 0.82, recall = 0.81, and *F*-measure = 0.81); it showed good results for identifying normal (sensitivity = 0.84, specificity = 0.94), MCI (sensitivity = 0.62, specificity = 0.93), VMD (sensitivity = 0.72, specificity = 0.93), and dementia (sensitivity = 0.92, specificity = 0.95).

## Figures and Tables

**Table 1:** Comparison of demographic data among the groups with different stages of cognitive impairment.

Group	CDR 0	CDR 0.5 (MCI)	CDR 0.5 (VMD)	CDR ≥ 1	*F*/*x*^2^	*p*

*N*	51	113	98	265		
Age, year (mean (SD))	68.1 (10.7)	71.8 (9.3)	76.1 (8.9)	78.9 (9.5)	30.772	<0.001*
Female, *N* (%)	24 (47.1)	55 (48.7)	59 (60.2)	156 (58.9)	5.689	0.128
Education, year (mean (SD))	6.9 (5.1)	6.4 (4.5)	4.4 (4.0)	4.5 (4.5)	8.452	<0.001**
MoCA, mean (SD)	21.1 (7.1)	18.0 (5.6)	11.1 (5.1)	7.2 (3.9)	202.176	<0.001*
CASI, mean (SD)	85.5 (11.3)	78.3 (10.1)	63.5 (14.0)	47.7 (15.1)	202.478	<0.001*
IADL, mean (SD)	8.0 (0.0)	7.3 (1.2)	6.0 (1.5)	2.7 (2.0)	314.797	<0.001*
NPI-sum, mean (SD)	3.0 (4.1)	5.6 (6.8)	6.1 (7.3)	9.7 (10.5)	12.386	<0.001***

CDR: Clinical Dementia Rating Scale; MCI: mild cognitive impairment; VMD: very mild dementia; N: number of participants; MoCA: Montreal Cognitive Assessment; IADL: Instrumental Activities of Daily Living; NPI-sum: sum score of Neuropsychiatric Inventory.

* Post hoc analysis showed CDR 0 < MCI < VMD < CDR≧1

** post hoc analysis showed CDR 0 = MCI > VMD = CDR≧1

*** post hoc analysis showed CDR 0 = MCI = VMD < CDR ≥ 1.

**Table 2:** The optimal model parameters.

Algorithm	Model parameters	Value

Random Forest	class_weight	Balanced
	max_depth	20
	n_estimators	20
	random_state	2018

AdaBoost	class_weight	Balanced
	base_estimator	Logistic regression
	Algorithm	SAMME
	n_estimators	10
	random_state	2018

LogitBoost	Classifier-maxDepth	RandomForest-5

MLP	hidden_layer_sizes	3
	random_state	2018

**Table 3:** Overall performance of the diagnostic models.

Algorithm	Accuracy	Precision	Recall	*F*-measure

Random Forest	0.86	0.85	0.86	0.85
AdaBoost	0.83	0.83	0.83	0.82
LogitBoost	0.81	0.81	0.81	0.80
MLP	0.87	0.87	0.87	0.87
Naive Bayes	0.87	0.88	0.87	0.87
SVM	0.87	0.86	0.87	0.86

Results were obtained by using all the 37 features.

**Table 4:** Overall performance of the diagnostic models.

Algorithm	Feature selection	Accuracy	Precision	Recall	*F*-measure

Random Forest	Relief	0.78	0.80	0.78	0.78
	Information Gain	0.78	0.79	0.78	0.78
	Random Forest	0.76	0.77	0.76	0.76

AdaBoost	Relief	0.77	0.78	0.77	0.77
	Information Gain	0.77	0.78	0.77	0.77
	Random Forest	0.76	0.76	0.76	0.76

LogitBoost	Relief	0.80	0.75	0.80	0.76
	Information Gain	0.78	0.73	0.78	0.74
	Random Forest	0.76	0.77	0.76	0.74

MLP	Relief	0.81	0.75	0.81	0.77
	Information Gain	0.79	0.73	0.79	0.75
	Random Forest	0.78	0.76	0.78	0.76

Naïve Bayes	Relief	0.79	0.74	0.79	0.75
	Information Gain	0.81	0.82	0.81	0.81
	Random Forest	0.77	0.80	0.77	0.78

SVM	Relief	0.80	0.74	0.80	0.76
	Information Gain	0.79	0.73	0.79	0.75
	Random Forest	0.76	0.74	0.76	0.75

Results were obtained after using the feature selection.

**Table 5:** Performance of the diagnostic models in the classification of normal, MCI, VMD, and dementia.

Algorithm	Class	Precision	Sensitivity	Specificity	*F*-measure

Random Forest	Normal	0.56	0.88	0.93	0.69
	MCI	0.70	0.57	0.93	0.62
	VMD	0.68	0.54	0.94	0.60
	Dementia	0.91	0.95	0.90	0.93

AdaBoost	Normal	0.55	0.84	0.93	0.67
	MCI	0.74	0.54	0.95	0.63
	VMD	0.63	0.55	0.93	0.59
	Dementia	0.89	0.94	0.88	0.92

LogitBoost	Normal	0.56	0.56	0.98	0.67
	MCI	0.73	0.73	0.89	0.65
	VMD	0.77	0.77	0.87	0.47
	Dementia	0.83	0.83	0.98	0.90

MLP	Normal	0.77	0.84	0.74	0.80
	MCI	0.65	0.74	0.89	0.69
	VMD	0.57	0.37	0.94	0.45
	Dementia	0.88	0.93	0.87	0.90
Naïve Bayes	Normal	0.56	0.84	0.94	0.67
	MCI	0.75	0.62	0.93	0.68
	VMD	0.70	0.72	0.93	0.71
	Dementia	0.95	0.92	0.95	0.93

SVM	Normal	0	0	1	0
	MCI	0.60	0.96	0.83	0.74
	VMD	0.85	0.56	0.98	0.67
	Dementia	0.91	0.97	0.90	0.94

## Data Availability

All relevant data are within the paper.
